# A Prospective Population-Based Study of Chimeric Antigen Receptor T-Cell Therapy for Patients with Diffuse Large B-Cell Lymphoma

**DOI:** 10.3390/curroncol33060366

**Published:** 2026-06-18

**Authors:** Lee Mozessohn, Pierre J. A. Villeneuve, Nibene H. Somé, Rebecca E. Mercer, Lisa Masucci, Tom Kouroukis, Christopher Bredeson, Suriya Aktar, Qi Guan, Anca Prica, Christine I. Chen, Danielle Rodin, Matthew C. Cheung, Munaza Chaudhry, Scott Gavura, Cassandra McKay, William W. L. Wong, Kelvin K. W. Chan

**Affiliations:** 1 Department of Medicine, University of Toronto, Toronto, ON M5S 3H2, Canadakelvin.chan@sunnybrook.ca (K.K.W.C.); 2 ICES, Toronto, ON M4N 3M5, Canada; 3 Odette Cancer Centre, Sunnybrook Health Sciences Centre, 2075 Bayview Avenue, Toronto, ON M4N 3M5, Canada; 4 Sunnybrook Research Institute, Toronto, ON M4N 3M5, Canada; rebecca.mercer@sunnybrook.ca; 5 The Ottawa Hospital, Ottawa, ON K1H 8L6, Canada; 6 Cancer Care Ontario, Ontario Health, Toronto, ON M5G 2M9, Canada; 7 Institute of Health Policy, Management and Evaluation, University of Toronto, Toronto, ON M5T 3M6, Canada; 8 Department of Epidemiology and Biostatistics, Western University, London, ON N6G 2M1, Canada; 9 Canadian Centre for Applied Research in Cancer Control (ARCC), Toronto, ON M4N 3M5, Canada; 10 University Health Network, Toronto, ON M5G 2C4, Canada; 11 Hamilton Health Sciences, McMaster University, Hamilton, ON L8N 3Z5, Canada; 12 Ottawa Hospital Research Institute, Ottawa, ON K1Y 4E9, Canada; 13 Princess Margaret Cancer Centre, Toronto, ON M5G 2C4, Canada; 14 Radiation Medicine Program, Princess Margaret Cancer Centre, Toronto, ON M5G 2C4, Canada; 15 Department of Radiation Oncology, University of Toronto, Toronto, ON M5G 2C4, Canada; 16 School of Pharmacy, University of Waterloo, Waterloo, ON N2G 1C5, Canada

**Keywords:** chimeric antigen receptor T-cell therapy, diffuse large B-cell lymphoma, real-world evidence

## Abstract

Chimeric antigen receptor (CAR) T-cell therapy is a new standard of care for patients with diffuse large B-cell lymphoma (DLBCL); however, studies including healthcare resource utilization (HRU) during routine care are lacking. Accordingly, a population-based study was conducted using linked administrative databases from Ontario, Canada. Patients with DLBCL that failed ≥2 lines of systemic therapy were included. Cox proportional hazard models estimated associations between covariates and overall survival (OS). Logistic, binomial and Poisson regression explored associations between covariates with toxicity and HRU. We identified 308 patients enrolled to receive CAR T-cell therapy of which 255 patients received CAR T-cells (mean age 59 years; 39% female). From the date of CAR T-cell infusion, the median OS was 25.0 months (95% CI, 21.6–28.1 months). Cytokine release syndrome and immune effector cell-associated neurotoxicity syndrome data were available for 155 patients and were reported in 135 (87.1%) and 42 (27.1%) patients, respectively. Of those that received CAR-T cells, 172 patients (67%) were hospitalized with a median length of stay of 5 days (IQR, 0–20) and 243 (95%) had an emergency department visit without hospitalization. Our prospective population-based study demonstrates comparable efficacy and safety of CAR T-cell therapy in the real-world to the pivotal trials and highlights this as an efficacious and relatively safe treatment option for patients with DLBCL in routine clinical care.

## Simple Summary

Chimeric antigen receptor (CAR) T-cell therapy is a standard treatment for relapsed/refractory diffuse large B-cell lymphoma (DLBCL). Despite widespread use, real-world studies, including healthcare utilization, are lacking. Using administrative databases in Ontario, Canada, researchers examined survival, toxicity and healthcare utilization. They demonstrated comparable efficacy and safety of CAR T-cell therapy in routine clinical care to the pivotal clinical trials. Further, healthcare utilization was similar to what has been reported in other registry studies. This study highlights CAR T-cell therapy as a relatively safe treatment option for patients with DLBCL in the real-world and emphasizes the need for the incorporation of health technology assessments in future studies.

## 1. Introduction

Diffuse large B-cell lymphoma (DLBCL) is the most common lymphoma accounting for almost 30% of all non-Hodgkin’s lymphoma [1]. Despite the majority of patients achieving a cure with frontline R-CHOP (rituximab, cyclophosphamide, doxorubicin, vincristine, and prednisone), the prognosis is poor for patients who are either refractory to frontline chemoimmunotherapy or relapse after autologous stem cell transplantation (ASCT) [2,3]. Indeed, patients with refractory disease have a complete response rate of 7% to next line of therapy with an overall survival (OS) of 6.3 months [4].

The introduction of chimeric antigen receptor (CAR) T-cell therapy has transformed the management of patients with relapsed and refractory (R/R) DLBCL. Tisagenlecleucel (tisa-cel) and axicabtagene ciloleucel (axi-cel) were the first two CD19 CAR T-cell products approved by Health Canada for patients with DLBCL ineligible for ASCT after two or more lines of therapy or with disease progression following ASCT. In the ZUMA-1 (axi-cel) and JULIET (tisa-cel) pivotal trials evaluating CAR T-cells for patients with R/R DLBCL, these therapies have been associated with complete response (CR) rates of up to 54% [5,6], some of which were durable. Longer-term follow-up demonstrated sustained responses in 31% of patients and a median OS of 25.8 months after 5 years of follow-up for axi-cel [7] and a median OS not reached for those treated with tisa-cel and achieving a CR with a median follow-up of over 3 years [8]. There are however challenges with the administration of CAR T-cell therapy, including the management of distinct toxicities, including cytokine release syndrome (CRS) and immune effector cell-associated neurotoxicity syndrome (ICANS) and the need for specialized care, challenging bridging therapy for the rapidly growing disease, and economic considerations [1].

The confirmation of efficacy and toxicity of CAR T-cell therapy in the real-world is critical given the high cost and resource requirements for its administration. Indeed, several studies based on real-world data have confirmed the survival, durable responses, and toxicity reported in the pivotal trials [9,10,11,12,13]. These studies were however not without limitations, including retrospective data collection, registry studies, or were from a single center and did not consider healthcare utilization. We thus conducted a prospective population-based study of all patients treated with publicly funded CAR T-cell therapy for DLBCL according to ZUMA-1 and JULIET inclusion criteria in routine clinical care with the allowance of bridging therapy to determine the association between patient characteristics and OS, toxicity, and healthcare resource utilization (HRU).

## 2. Methods

### 2.1. Patients

The study cohort comprised Ontario residents ≥18 years old with DLBCL ineligible for ASCT after two or more lines of therapy or with disease progression following ASCT proceeding with publicly funded commercially available CAR T-cell therapy from September 2019 to 30 June 2023, and followed to 31 December 2023, or the date of death, whichever occurred first. Patients with primary mediastinal B-cell lymphoma (PMBL), high grade B-cell lymphoma with or without MYC and BCL2 rearrangements, other aggressive B-cell lymphoma histologies (excluding Burkitt lymphoma) or DLBCL transformed from follicular lymphoma were also included. At the time of CAR T-cell therapy enrollment, patients were required to have adequate organ function, including renal, hepatic, pulmonary, cardiac, bone marrow (see Supplementary Materials File S1 for details) and an adequate performance status (Karnofsky Performance Status > 70; KPS). Bridging therapy, including systemic treatment and radiation, was allowed following cell collection as per the JULIET trial [5]. Those with active central nervous system (CNS) disease, previous treatment with CAR T-cell therapy or active uncontrolled infections were excluded.

All permanent residents of Ontario are covered by a single-player healthcare system with universal health care access where patients who meet eligibility criteria receive cancer therapies without deductibles or copayments. Consequently, we are able to capture the entire population of patients in Ontario treated with CAR T-cell therapy. Ontario Health (Cancer Care Ontario) (OH [CCO]) is an agency created by the Government of Ontario with a mandate to connect, coordinate and transform the province’s health system. OH (CCO)’s CAR T-cell Therapy Program mandates the completion of CAR T-cell therapy enrollment forms, including patient demographic and baseline characteristics, to determine funding eligibility. Treating centers are then required to prospectively complete forms created by the Center for International Blood and Marrow Transplant Research (CIBMTR) to denote treatment efficacy and toxicity. These forms are then submitted to OH (CCO) for program evaluation and were used for this study. In Ontario, CAR T-cell therapy is centralized with administration at select specialized centers. OH (CCO) is designated a “prescribed entity” for the purposes of section 45(1) of the Personal Health Information Protection Act of 2004. As a prescribed entity, OH (CCO) is authorized to collect personal health information from health information custodians without the consent of the patient, and to use such personal health information for the purpose of analysis or compiling statistical information with respect to the management, evaluation, or monitoring of the allocation of resources to or planning for all or part of the health system, including the delivery of services. Because this study is in compliance with privacy regulations, ethics review was not required.

### 2.2. Data Sources

Demographic variables and baseline characteristics, including age and sex were identified from the CAR T-cell Therapy Program enrollment forms with treatment data from OH (CCO)’s CAR T-cell therapy dataset. Income quintile was assigned from Census data according to residential postal code at the neighborhood level (lowest quintile represents lowest household income). Radiation therapy was obtained from the Cancer Activity Level Reporting (ALR) dataset. Toxicity data was ascertained from the CIBMTR forms and the date of death extracted from the Office of the Registrar General Database (ORGD). Hospitalization and ED visits were determined using International Classification of Diseases, Tenth Revision (ICD-10) codes in Canadian Institute for Health Information-Discharge Abstract Database (CIHI-DAD) or the National Ambulatory Care Reporting System (NACRS), respectively.

### 2.3. Outcomes

Our primary outcome was OS defined from the date of CAR T-cell infusion to the date of death or censored at the end of the study period (‘per-protocol’), consistent with the ZUMA-1 and JULIET studies [5,6]. We also separately examined OS in all patients enrolled to receive CAR T-cell therapy defined from the date of enrollment to the date of death or censored at the end of the study period (‘intention-to-treat’). Secondary outcomes included incidence and grade of CRS and ICANS as per the American Society for Transplantation and Cellular Therapy [14], tocilizumab usage, and admissions for febrile neutropenia based on previously published methods for the ‘per-protocol’ population [15]. For the ‘per-protocol’ population, we also examined hospitalization (excluding admission for CAR T-cell infusion) and intensive care unit (ICU) admission, including length of stay (LOS) and unplanned emergency department (ED).

### 2.4. Covariates

Covariates included age, sex, Karnofsky Performance Score (KPS), income quintile, and rurality (population of <10,000). Charlson Comorbidity Index (CCI) was used to characterize comorbidity at the time of CAR T-cell therapy enrollment and excluded cancer-related International Classification of Diseases (ICD)-10 codes [16,17]. Radiotherapy prior to CAR T-cell therapy enrollment was obtained from the Cancer Activity Level Reporting (ALR) database and was not counted as a line of therapy to determine CAR T-cell eligibility.

### 2.5. Statistical Analysis

Descriptive statistics of baseline characteristics are presented using means with standard deviations, medians with interquartile ranges (IQR), or frequency with percentages where appropriate. We used univariate and multivariable Cox proportional hazard models to estimate the hazard ratios (HRs) and 95% confidence intervals (CIs) of CAR T-cell therapy on survival with the Kaplan–Meier method to generate survival curves. Variables included in the multivariable models were age, sex, rurality, prior radiotherapy, time from lymphoma diagnosis to CAR T-cell therapy enrolment, KPS score, income quintile, CCI score, treating center and lymphoma diagnosis (DLBCL, primary mediastinal B-cell lymphoma [PMBL], transformed lymphoma). For binary outcomes including tocilizumab use, presence of CRS/ICANS and ICU admission, logistic regression was used and expressed as odds ratios (ORs). The association between covariates and count outcomes were analyzed using Poisson and negative binomial regressions. Pearson Chi-squared statistics were used to test for over-dispersion, which determined the model with the best fit. Consequently, the duration of CRS (days) and LOS for the CAR T-cell infusion hospitalization were explored using negative binomial regression whereas duration of ICANS (days) and ICU LOS were determined with Poisson regression and expressed as rate ratios (RR). For all analyses, a 2-tailed *p* < 0.05 was considered statistically significant. Analyses were conducted using SAS v.9.4 (SAS Institute Inc., Cary, NC, USA).

## 3. Results

### 3.1. Study Population

There were 308 patients (‘intention-to-treat’) enrolled to receive CAR T-cell therapy between 1 January 2020 and 30 June 2023, of which 255 received CAR T-cells (‘per-protocol’) with 118 (46.3%) receiving tisa-cel and 137 (53.7%) axi-cel (Table 1 summarizes the baseline characteristics for the ‘per-protocol’ population). Most patients had DLBCL (73%) with the remaining having DLBCL arising from follicular lymphoma (21%) and PMBL (5%). Mean age (standard deviation) was 59 (±13) years and 39% were female. Systemic bridging therapy was administered to 75 patients (29.4%) with polatuzumab-bendamustine-rituximab (31 patients; pola-BR) as the most common regimen. The median follow-up was 11.1 months (95% CI, 9.3 to 13.2 months).

### 3.2. Overall Survival

**Figure 1:**
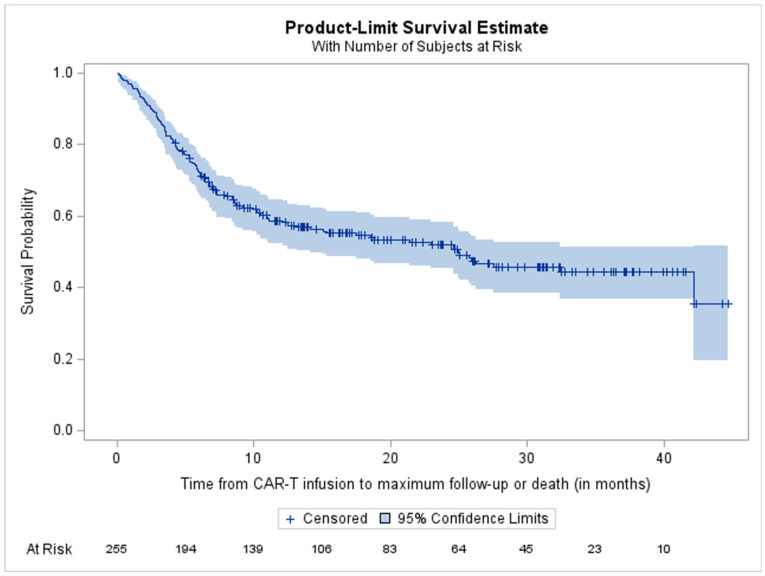
Overall survival.

From the date of CAR T-cell infusion until 30 June 2023, 124 patients (48.6%) died (‘per-protocol’ population; Figure 1). The median OS from the date of CAR T-cell infusion was 25.0 months (95% CI, 21.6 to 28.1 months) compared with 15.2 months (95% CI, 11.1 to 26.3 months) from the date of CAR T-cell enrollment (‘intention-to-treat’ population). For the ‘per-protocol’ population, the 12-month and 24-month survival was 58.5% (95% CI, 52.4 to 64.7%) and 52.4% (95% CI, 45.8 to 58.9%), respectively. On multivariable analysis, radiation (adjusted hazard ratio; aHR 1.71; 95% CI, 1.16 to 2.53, *p* = 0.01) prior to CAR T-cell enrollment was associated with worse survival whereas KPS greater than 70 (aHR 0.42; 95% CI, 0.20 to 0.89, *p* = 0.02) was associated with improved survival. None of the other covariates were associated with survival (Table 2). The 30-day mortality rate from the date of CAR T-cell infusion was 4.6%.

### 3.3. Safety Outcomes and Healthcare Utilization

CRS and ICANS data were available for 155 patients of which, CRS was seen in 135 patients (87.1%) with a median duration of 4 days (IQR, 3 to 6 days) and ICANS in 42 patients (27.1%) with a median duration of 5 days (IQR, 2 to 8 days). Tocilizumab was administered to 180 patients (70.6%) with a median total dose of 1000 mg (IQR, 645 mg to 1530 mg). None of the covariates were associated with an increased odds of CRS whereas there was an association between CAR T-cell product (axi-cel vs. tisa-cel: odds ratio; OR 4.18; 95% CI, 1.49–11.70, *p* = 0.007) and CAR T-cell therapy enrollment site with ICANS though early in the program, there was variation in the CAR T-cell product availability at different sites (Table 3). There was also an increased odds of ICANS with income quintile 3 versus quintiles 4 and 5.

The median length of hospitalization for the CAR T-cell infusion was 15 days (IQR, 10 to 22 days). A total of 30 patients (11.8%) required ICU admission with a median LOS of 3 days (IQR, 2 to 7 days). None of the predictors were associated with an increased odds of ICU admission whereas CAR T-cell product (axi-cel), previous radiation and income quintile (quintile 1 and 2 versus quintile 4 and 5) were associated with a longer length of ICU stay (Table 4). Following CAR T-cell infusion, 172 patients (67.5%) were hospitalized (excluding the hospitalization for CAR T-cell infusion) with a median LOS of 5 days (IQR, 0 to 20), 243 (95.3%) had an ED visit not resulting in a hospital admission and 85 (33.3%) had both an ED visit not resulting in a hospitalization and hospital admission. There were 35 (13.7%) of patients that had febrile neutropenia.

## 4. Discussion

In this prospective population-based study that comprehensively captures all publicly funded CAR T-cell therapy administration in Ontario, Canada for patients with relapsed/refractory DLBCL, we demonstrate that the efficacy and safety of CAR T-cell therapy in the real-world is comparable to the reported outcomes in clinical trials. The median OS of 25.0 months and CRS rate of 87% in our population is similar to ZUMA-1 though our rates of ICANS were lower (27% versus 64%), which may reflect that almost half of the patients in our cohort received tisa-cel [7,12]. By comparison, JULIET reported a CRS and ICANS rate of 57% and 20%, respectively [8]. Our results highlight that CAR T-cell therapy remains both an efficacious and relatively safe treatment option for this patient population in routine clinical care.

The reported OS in our study is consistent with other real-world studies. For example, a study of patients treated with commercial axi-cel from the CIMBTR registry reported a median OS of 21.8 months though 57% of patients were considered ZUMA-1 ineligible [9]. The most common reasons for ineligibility were comorbidities, including other prior malignancies and poor performance status whereas our study aligned with the inclusion criteria for ZUMA-1. Similarly, our 24-month OS of 52% compared favorably with an additional CIMBTR registry study of tisa-cel where they reported a 24-month OS of 44% and improved efficacy for patients with DLBCL (versus high-grade B-cell lymphoma), complete response before CAR T-cell infusion, previous stem cell transplant, and normal lactate dehydrogenase (LDH), whereas we found that radiation prior to CAR T-cell enrollment was associated with an inferior OS—likely a surrogate for higher risk or refractory disease though we cannot exclude increased toxicity as an additional possible explanation [18]. Further, they reported an increased odds of CRS and ICANS in patients with poor performance status, elevated LDH, ≥3 lines of therapy, and fludarabine lymphodepleting chemotherapy, whereas none of our predictors were associated with the occurrence of CRS. Our finding of differences in ICANS rates based on enrollment site may reflect the variability in CAR T-cell product availability at the start of the program as some sites preferentially selected one product over another with higher ICANS rates previously reported with axi-cel consistent with our findings [12]. Given the marginal association between patient characteristics with efficacy and toxicity in our study and others, our findings suggest these characteristics should not preclude the receipt of CAR T-cell therapy and that close vigilance for complications is needed for all patients.

Our healthcare utilization findings add to the growing body of literature suggesting high HRU for patients undergoing CAR T-cell therapy with 54% of patients needing hospital re-admission with a median LOS of 5 days. For example, recent studies using claims data report a mean total inpatient hospital stay that ranged from 16 to 22 days with a readmission rate of 20% to 37% in the immediate post-infusion period (within 30 days to 3 months after CAR-T infusion) and high total health expenditures though there may be reduced HRU compared to historical controls [19,20,21]. Our ICU admission rate compares favorably to other studies reporting rates of ICU transfer for severe toxicity that ranged from 27% to 35% with the inclusion of patients with follicular lymphoma, multiple myeloma and acute lymphoblastic leukemia though a similar median LOS of 4 days [22,23]. Lower income was associated with a longer ICU LOS and highlights the complex interplay between marginalization and outcomes in patients with DLBCL in a single-payer healthcare system [24]. Although we did not evaluate disparities in access to CAR T-cell therapy, other studies report decreased access in those residing in lower income areas and those with Medicare or lack of insurance emphasizing the need to include these factors in understanding CAR T-cell therapy access and outcomes [25,26].

Our study has several important limitations. Given that this was a population-based analysis, we did not have granular information on disease risk (e.g., LDH), which is a known prognostic factor in this patient population [18]. We were unable to determine the cause of death (i.e., due to disease progression versus treatment complications) though our 30-day mortality rate was low (less than 5%). We were also unable to obtain the CAR T-cell therapy response, which would have allowed for an assessment of progression- and disease-free survival as well as grade of toxicity. Further, our study was a single-arm design and did not include a comparison group to better contextualize the efficacy outcomes though historically, the outcomes for patients with refractory DLBCL are poor [4]. We also did not evaluate costs of care which is of particular importance when considering healthcare expenditures at a population-level, including considerable out-of-pocket costs, and should be explored in future studies [27]. Strengths of our study include its population-based design in a single-payer healthcare system with universal access and evaluation of HRU.

In conclusion, our population-based study of patients receiving publicly funded commercially available CAR T-cell therapy demonstrates comparable efficacy to the landmark clinical trials and toxicity similar to what has been reported in other real-world registries. Our findings reinforce this therapy as a standard of care in routine clinical practice and highlight the need for the incorporation of health technology assessments in future studies.

## Figures and Tables

**Table 1:** Baseline Characteristics of the ‘Per-protocol’ Population.

	Total (N = 255)
**Age at CAR T-cell Enrollment, years**	
Mean (±SD)	58.9 ± 13.2
**Female, No. (%)**	100 (39.2)
**Rural Residence, No. (%)**	44 (17.3)
**Income Quintile, No. (%)**	
1 (lowest) and missing	36 (14.1) ^a^
2	46 (18.0)
3	46 (18.0)
4	61 (23.9)
5	66 (25.9)
**Charlson Comorbidity Score, No. (%)**	
0	202 (79.2)
1 to 2	31 (12.2)
≥3	22 (8.6)
**Karnofsky Performance Score, No. (%)**	
≤70 and missing	15 (5.9) ^a^
>70	240 (94.1)
**Diagnosis, No. (%)**	
DLBCL	186 (72.9)
DLBCL arising from follicular lymphoma	53 (20.8)
PMBL	12 (4.7)
**Radiation Prior to CAR T-cell Enrollment, No. (%)**	78 (30.6)
**Time from initial diagnosis to CAR T-cell Enrollment, years**	
Mean (±SD)	3.0 (4.4)
**Index Year, No. (%)**	
2020	46 (18.0)
2021	81 (31.8)
2022	86 (33.7)
2023	42 (16.5)
**CAR T-cell Enrollment Site, No. (%)**	
Site 1	86 (33.7)
Site 2	58 (22.8)
Site 3	111 (43.5)

Abbreviations: SD, standard deviation; DLBCL, diffuse large B-cell lymphoma; PMBL, primary mediastinal B-cell lymphoma. ^a^ In accordance with data policy, patients with missing income quintile and Karnofsky Performance Score cannot be reported separately to avoid re-identification.

**Table 2:** Multivariable model examining the association between predictors and overall survival.

Predictor	Reference	aHR	95% CI	*p*-Value
**Sex (male)**		1.27	0.87–1.86	0.22
**Age (>60 years)**	≤60 years	1.27	0.87–1.86	0.22
**Rural residence (yes)**		0.71	0.42–1.19	0.20
**Radiation prior to CAR T-cell enrollment (yes)**		1.71	1.16–2.53	0.01
**Time from initial diagnosis to CAR T-cell infusion (years)**		0.96	0.91–1.01	0.09
**Karnofsky Performance Score > 70**	KPS ≤ 70	0.42	0.20–0.89	0.02
**Treatment center**				
Site 1	Site 2	0.93	0.52–1.66	0.80
Site 3	Site 2	0.96	0.58–1.59	0.87
**Charlson Comorbidity Score**				
CCS score (1 to 2)	CCS score 0	0.78	0.43–1.41	0.42
CCS score (≥3)	CCS score 0	0.69	0.34–1.42	0.32
**Income quintile (quintile 1 = lowest)**				
Income quintile 1 and 2	Quintile 4 and 5	0.86	0.59–1.34	0.56
Income quintile 3	Quintile 4 and 5	0.89	0.53–1.49	0.66
**Diagnosis**				
DLBCL arising from FL	DLBCL	0.80	0.51–1.27	0.34
PMBL	DLBCL	1.04	0.43–2.51	0.93
**CAR T-cell product (axi-cel)**	Tisa-cel	1.04	0.65–1.67	0.86

Abbreviations: aHR, adjusted hazard ratio; CI, confidence interval; KPS, Karnofsky Performance Score; CCS, Charlson Comorbidity Score; DLBCL, diffuse large B-cell lymphoma; FL, follicular lymphoma; PMBL, primary mediastinal B-cell lymphoma.

**Table 3:** Multivariable model examining the association between predictors, CRS and ICANS (any grade).

		CRS		ICANS	
Predictor	Reference	OR (95% CI)	*p*-Value	OR (95% CI)	*p*-Value
**Sex (male)**		1.24 (0.45–3.46)	0.68	1.77 (0.73–4.21)	0.20
**Age (>60 years)**		0.51 (0.16–1.64)	0.26	1.66 (0.69–3.99)	0.26
**Rural residence (yes)**		--	0.95	0.69 (0.21–2.29)	0.54
**Radiation prior to CAR T-cell enrollment (yes)**		0.84 (0.29–2.50)	0.76	1.18 (0.47–2.92)	0.73
**Time from initial diagnosis to CAR T-cell infusion (years)**		1.03 (0.89–1.15)	0.84	0.99 (0.89–1.10)	0.83
**Karnofsky Performance Score > 70**	KPS ≤ 70	0.99 (0.10–9.72)	0.99	2.04 (0.19–22.34)	0.56
**Treatment center**					
Site 1	Site 2	0.45 (0.09–2.37)	0.34	0.40 (0.13–1.26)	0.18
Site 3	Site 2	0.56 (0.08–3.78)	0.55	0.19 (0.05–0.64)	0.008
**Charlson Comorbidity Score**					
CCS score (1 to 2)	CCS score 0	0.54 (0.12–2.43)	0.42	1.91 (0.52–7.05)	0.33
CCS score (≥3)	CCS score 0	0.60 (0.10–3.56)	0.58	2.47 (0.50–12.17)	0.27
**Income quintile (quintile 1 = lowest)**					
Income quintile 1 and 2	Quintile 4 and 5	0.86 (0.27–2.77)	0.80	1.13 (0.42–3.00)	0.81
Income quintile 3	Quintile 4 and 5	0.77 (0.20–3.01)	0.71	3.30 (1.15–9.46)	0.03
**CAR T-cell product** **(axi-cel)**	Tisa-cel	1.53 (0.38–6.22)	0.56	4.18 (1.49–11.70)	0.007

Abbreviations: CRS, cytokine release syndrome; ICANS, immune effector cell-associated neurotoxicity syndrome; OR, odds ratio; CI, confidence interval; KPS, Karnofsky Performance Score; CCS, Charlson Comorbidity Score.

**Table 4:** Multivariable model examining the association between predictors, ICU admission and ICU length of stay.

		ICU Admission		ICU Length of Stay	
Predictor	Reference	OR (95% CI)	*p*-Value	RR (95% CI)	*p*-Value
**Sex (male)**		0.69 (0.30–1.59)	0.39	1.38 (0.68–2.78)	0.37
**Age (>60 years)**		1.26 (0.53–2.97)	0.60	1.52 (0.80–2.89)	0.20
**Rural residence (yes)**		1.72 (0.63–4.66)	0.29	0.72 (0.30–1.74)	0.47
**Radiation prior to CAR T-cell enrollment (yes)**		0.49 (0.17–1.46)	0.20	3.28 (1.12–9.62)	0.03
**Time from initial diagnosis to CAR-T cell infusion (years)**		0.91 (0.79–1.06)	0.24	0.93 (0.84–1.03)	0.18
**Karnofsky Performance Score > 70**	KPS ≤ 70	0.52 (0.09–2.84)	0.45	3.37 (0.55–20.62)	0.19
**Treatment center**					
Site 1	Site 2	0.50 (0.14–1.79)	0.29	0.51 (0.23–1.14)	0.10
Site 3	Site 2	0.55 (0.19–1.63)	0.28	0.89 (0.36–2.18)	0.79
**Charlson Comorbidity Score**					
CCS score (1 to 2)	CCS score 0	1.31 (0.32–5.41)	0.71	1.05 (0.43–2.55)	0.92
CCS score (≥3)	CCS score 0	1.78 (0.43–7.42)	0.43	0.50 (0.11–2.22)	0.36
**Income quintile (quintile 1 = lowest)**					
Income quintile 1 and 2	Quintile 4 and 5	0.83 (0.32–2.15)	0.70	2.29 (1.14–4.61)	0.02
Income quintile 3	Quintile 4 and 5	0.75 (0.22–2.55)	0.64	1.93 (0.85–4.40)	0.12
**Diagnosis**					
DLBCL arising from FL	DLBCL	0.37 (0.10–1.35)	0.13	1.16 (0.41–3.30)	0.78
PMBL	DLBCL	0.73 (0.08–6.52)	0.77	2.54 (0.57–11.28)	0.22
**CAR T-cell product** **(axi-cel)**	Tisa-cel	0.69 (0.24–2.01)	0.49	0.42 (0.20–0.88)	0.02

Abbreviations: ICU, intensive care unit; OR, odds ratio; CI, confidence interval; RR, rate ratio; KPS, Karnofsky Performance Score; CCS, Charlson Comorbidity Score; DLBCL, diffuse large B-cell lymphoma; FL, follicular lymphoma; PMBL, primary mediastinal B-cell lymphoma.

## Data Availability

Ontario Health is prohibited from making the data used in the research publicly accessible if it includes potentially identifiable personal health information and/or personal information as defined in Ontario law, specifically the Personal Health Information Act (PHIPA) and the Freedom of Information and Protection of Privacy Act (FIPPA). Upon request, data de-identified to a level suitable for public release may be provided.

## Supplementary Materials

The following supporting information can be downloaded at: https://www.mdpi.com/article/10.3390/curroncol33060366/s1, File S1: Adequate organ function definitions.
