# Supplementary material for: A Prospective Population-Based Study of Chimeric Antigen Receptor T-Cell Therapy for Patients with Diffuse Large B-Cell Lymphoma
Source: Curr Oncol. 2026 Jun 18;33(6):366. doi: 10.3390/curroncol33060366 (PMC13297695; doi:10.3390/curroncol33060366)
Supplement: Supplementary file 1 [file curroncol-33-00366-s001.zip › curroncol-4259413-supplementary.pdf]

File S1:Adequate organ function definitions.

Adequate organ function at the time of CAR T-cell therapy enrollment was defined as:

1. Renal function
    - Serum creatinine <1.6 mg/dL [<141.44 ummol/L])
    - Estimated glomerular filtration rate (eGFR) >45 ml/min/1.73 m^2
2. Hepatic function
    - ALT or AST <3x upper limit of normal value
    - Bilirubin <2x upper limit of normal value
3. Pulmonary function
    - Pulse oxygenation >91% on room air
4. Cardiac function
    - Left ventricular ejection fraction >40%) confirmed by echocardiogram or multiple-gated acquisition (MUGA) scan or radionuclide angiography
5. Bone marrow function
    - Absolute neutrophil count >1x10^9/L
    - Absolute lymphocyte count (ALC) $\geq$ 0.1x10^9/L (if ALC below 0.1x10^9/L, still eligible but for apheresis to proceed, ALC must be at least 0.1x10^9/L)
    - Hemoglobin >80 g/L and/or transfusion dependent
    - Platelets >50x10^9/L
